# 基于手性有机框架材料制备气相色谱固定相的研究进展

**DOI:** 10.3724/SP.J.1123.2023.07021

**Published:** 2024-01-08

**Authors:** Suxin ZHOU, Yixin KUANG, Juan ZHENG, Gangfeng OUYANG

**Affiliations:** 1.中山大学化学学院, 广东 广州 510006; 1. School of Chemistry, Sun Yat-sen University, Guangzhou 510006, China; 2.中山大学化学工程与技术学院, 广东 珠海 519082; 2. School of Chemical Engineering and Technology, Sun Yat-sen University, Zhuhai 519082, China

**Keywords:** 对映异构体分离, 手性共价有机框架, 手性金属有机框架, 气相色谱, 综述, enantiomer separation, chiral covalent organic framework, chiral metal-organic framework, gas chromatography, review

## Abstract

对映异构体通常具有不同的药理学、毒理学和生理学性质,获得单一手性化合物对人类健康和环境的可持续发展均具有重要意义。目前,色谱是分离对映异构体的主要方式之一,色谱分离的关键在于固定相的选择。有机框架材料作为一类新兴的结晶多孔材料,具有结构高度有序、孔隙丰富、孔结构和尺寸可调及易于功能化等优点,在对映异构体的色谱分离方面受到广泛关注。通过后修饰或自下而上的合成策略,一系列具有高结晶度和丰富手性识别位点的有机框架材料已经被成功研制。基于动态涂覆或原位生长等方法,手性有机框架材料可被成功固定于气相色谱柱的内表面,从而实现多种对映异构体混合物的高分辨分离;与商用手性色谱柱相比,部分自制的手性有机框架材料色谱柱具有更优异的选择因子和分离度。本文首先介绍了有机框架材料在分离领域所展现出的优势,之后分别论述了手性有机框架材料的合成方式、相应色谱固定相的制备方法及手性有机框架材料对对映异构体的分离性能,最后总结了手性有机框架材料在未来手性材料领域的突出优势和面临的挑战。

## Abstract (en)

Enantiomers typically show different pharmacological, toxicological, and physiological properties. Thus, the preparation of enantiopure compounds is of great significance for human health and sustainable development. Compared with asymmetric catalysis, enantiomeric separation is simpler, faster, and more efficient; as such, it has become the preferred method for obtaining pure enantiomers. At present, enantiomeric separation methods mainly include chromatography, nanochannel membrane separation, selective adsorption, and recrystallization. In particular, gas chromatography (GC) plays an important role in enantioseparation because of its high sensitivity, excellent reproducibility, and outstanding processing capacity for various enantiomers. The stationary phase is key to the separation efficiency of GC, and more efficient, stable, and cost-effective materials that could serve as stationary phases are constantly being explored. Organic frameworks, such as covalent organic frameworks (COFs), metal-organic frameworks (MOFs), porous organic cages (POCs), metal-organic cages (MOCs), and hydrogen-bonded organic frameworks (HOFs), possess large specific surface areas, high porosities, tunable pore sizes, and easy functionalization, rendering them promising candidates for the separation of mixed analytes. Research has shown that the use of organic frameworks as stationary phases for GC results in excellent column efficiency and high resolution for various analytes, including *n*-alkanes, *n*-alcohols, polycyclic aromatic hydrocarbons, positional isomers, and organic fluorides. Furthermore, organic frameworks can be prepared as chiral stationary phases for GC by the intelligent introduction of a chiral moiety, thereby enabling the efficient separation of enantiomers. Synthetic strategies for chiral organic frameworks are primarily categorized as post-synthesis or bottom-up approaches. In general, the post-synthesis strategy can introduce various chiral sites to the framework; however, the distribution of chiral sites may not be uniform, and the ordered framework may be destroyed during the post-synthesis process. The bottom-up strategy allows for the uniform and precise distribution of chiral sites in the framework, but the synthesis of chiral monomers and the constraint between asymmetry and crystallinity limit its development. Chiral induction has been proposed as an alternative strategy for synthesizing chiral organic frameworks. The use of this strategy has led to the successful preparation of organic frameworks with abundant chiral sites and excellent crystallinity. Dynamic coating and in situ growth are the main approaches used to transform the as-prepared chiral organic frameworks into stationary phases. Notably, the in situ growth approach can yield chiral COF/MOF-coated capillary columns that provide high resolution for the separation of enantiomers with excellent repeatability and reproducibility. Nevertheless, owing to the slightly complex pretreatment process and the difficulty of synthesizing chiral organic frameworks, the in situ growth approach has not yet been widely applied. Owing to their excellent solvent processing performance, POCs, MOCs, and HOFs can be easily coated on the inner walls of columns to form membranes via dynamic or static coating. A series of enantiomers have been successfully separated and analyzed by immobilizing chiral COFs, MOFs, POCs, MOCs, and HOFs on GC capillary columns, demonstrating the great potential of chiral organic frameworks for enantiomeric separation. In general, the mechanisms by which chiral organic frameworks recognize enantiomers could be mainly categorized as van der Waals interactions, hydrogen bonding, *π*-*π* interactions, and size-exclusion effects. While molecular simulations can offer some insights into these recognition mechanisms, clarifying these mechanisms based on effective characterization remains challenging. In summary, organic frameworks show outstanding advantages for enantiomer separation. Given breakthroughs in synthetic strategies for chiral organic frameworks and the in-depth study of chiral recognition mechanisms, chiral organic frameworks may be expected to become an important aspect in the field of chiral materials, further realizing the large-scale analysis and production of chiral analytes. A total of 64 references, most of which are from the American Chemical Society, Springer Nature, Wiley Online Library, and Elsevier databases, are cited in this review.

对映异构体通常具有不同的药理学、毒理学和生理学效应,甚至完全相反^[1⇓-3]^。例如在医药领域,*S*-萘普生具有理想的治疗活性,而*R*-萘普生对人体有副作用;在食品领域,*S*,*S*-天冬甜素的甜度是方糖的200倍,而*R*,*R*-天冬甜素的味道偏苦^[1]^。因此,单一手性化合物的制备对人类健康和环境的可持续发展均具有重要意义。目前,获得单一手性化合物的方式主要有两种,一种是直接通过不对称催化方法制得^[4,5]^,另一种是通过分离对映异构体获得^[6,7]^。与不对称催化方法相比,对映异构体分离方法更加简单、快速、高效,且没有副产物,是一种环境友好和经济性高的方法,已成为获得单一手性化合物的主要方式^[6]^。为了实现高效的对映异构体分离,在过去几十年研究人员付出了巨大的努力。目前常见的对映异构体分离方式主要包括色谱分离^[8,9]^、膜分离^[10]^、选择性吸附^[11,12]^和重结晶^[13,14]^;其中,色谱分离具有简单、快速、可重复性好、灵敏度高等优点,且能够同时分离不同的对映异构体。对气相色谱而言,惰性气体的使用也能够避免由于溶剂效应等因素引起的其他问题,因此气相色谱十分适用于稳定的挥发性手性化合物的分离分析^[15]^。

对于色谱分离,固定相的选择是分离的关键,探索更高效、稳定、价格低廉的固定相材料是目前色谱分离领域的研究热点。作为新兴的结晶多孔材料,共价有机框架(covalent organic frameworks, COFs)、金属有机框架(metal-organic frameworks, MOFs)、多孔有机笼(porous organic cages, POCs)、金属有机笼(metal-organic cages, MOCs)和氢键有机框架(hydrogen-bonded organic frameworks, HOFs)等材料凭借其高度有序的框架结构、丰富的孔隙、可调的孔结构和尺寸及易功能化等优势已在分离领域引起了广泛的关注^[8⇓-10,16]^。值得注意的是,通过后修饰或自下而上的策略引入手性位点,可使有机框架材料具有手性分离能力,从而实现对映异构体的高效分离。

本文讨论了近几年来手性有机框架材料(包括手性COFs、手性MOFs、手性POCs、手性MOCs以及手性HOFs)的主要合成策略,阐述其作为气相色谱固定相在对映异构体分离中的研究进展,最后对手性有机框架材料作为气相色谱固定相的应用前景进行了展望。

## 1 有机框架材料在色谱分离中的应用

有机框架材料具有丰富的孔隙,且易功能化,其可通过一种或多种相互作用(如离子-偶极作用、氢键、空间位阻、*π-π*相互作用、范德华力等)选择性地吸附一种或几种分析物,从而实现混合物的分离^[6]^。目前,有机框架材料已在色谱分离领域吸引了大量的关注。例如,Yang等^[17]^在温和的条件下利用三醛基间苯三酚和联苯二胺缩合制备了球形的COF颗粒,之后将COF颗粒涂覆到气相色谱毛细管柱上,可用于分离烷烃、环己烷、苯、*α*-蒎烯、*β*-蒎烯和醇。此外,为了解决多晶COFs颗粒形状和尺寸不均一的问题,Zheng等^[18]^和Wang等^[19]^合成了两种单晶COFs颗粒,分别将其应用于高效液相色谱填充柱和气相色谱毛细管柱中,成功实现了多种同分异构体的有效分离;此外,通过比较不同粒径COF颗粒的分离效果发现,尺寸较大的COF颗粒具有较弱的尺寸排斥效应,且传质阻力高,导致其对异构体分离的分辨率和柱效降低。Fu等^[20]^基于1,3,5-三(4-甲酰基苯基)苯和联苯二胺缩合得到COF颗粒,并将COF颗粒固定在毛细管电色谱柱上,实现了对烷基苯、氯苯、酚类、苯胺类、氨基酸类和对羟基苯甲酸酯类混合物的分离。Zheng等^[21]^设计了一种氟功能化的球形COF颗粒,并将其作为固定相用于高效液相色谱中,所制备的色谱柱对多氟苯、甲基丙烯酸全氟烷基酯、卤代三氟甲苯等有机氟化物均表现出高的柱效和优异的分离度。我们课题组^[22]^也成功合成了一种MOF材料(metal azolate framework-5, MAF-5),并将其固定在气相色谱毛细管柱上,实现了对多环芳烃和有机氯农药的分离。由此可见,有机框架材料在色谱分离领域具有很大的应用潜力,通过将手性识别位点固定在有机框架材料骨架上制备成手性有机框架材料,有望进一步提高有机框架材料的分离能力,拓宽其应用范围。手性COFs、手性MOFs、手性POCs、手性MOCs以及手性HOFs等手性有机框架材料在色谱分离领域也受到了越来越多的关注。

## 2 手性共价有机框架

COFs是一类由C、H、O、N、B等元素通过共价键结合的多孔结晶材料,其孔隙丰富,密度低,且稳定性高。与其他多孔材料相比,COFs具有独特的结构和多样性的功能,在吸附^[23,24]^、催化^[25]^等领域都是十分有潜力的材料,在分离领域也受到了广泛的关注。通过对COFs微观结构的精细调控,COFs材料可拥有更高的比表面积^[26]^和结晶度^[26]^以及更适合的尺寸^[27]^、孔径^[28]^和极性^[29]^,从而提高其分离性能。在此基础上,将手性识别位点固定在COFs骨架上^[8,26]^,可使其具有手性功能,进而在分离领域表现出更突出的作用,尤其是对对映异构体的分离。虽然手性COFs在对映异构体的分离方面极具潜力,但结构稳定的手性COFs的合成仍存在一定的挑战,将手性COFs固定在气相色谱毛细管柱上也需要掌握相应的方法。在2.1~2.3节将对手性COFs的合成、手性COFs色谱柱的制备以及手性COFs在色谱分离中的应用逐一进行介绍。

### 2.1 手性共价有机框架材料的合成

将手性功能部分引入COFs的方法主要可以分为后合成(post-synthesis)和自下而上(bottom-up)两种方式,此外,通过手性诱导(chiral induction)也可以获得手性COFs。后合成是引入手性功能最常用的方法,可在原有COFs的基础上引入多种理想的手性中心。例如,Xu等^[30]^首先设计了具有高稳定性、高结晶度和高孔隙率的介孔COF(TPB-DMTP-COF),之后在TPB-DMTP-COF的基础上通过三组分系统,即同时加入1,3,5-三(4-氨基苯基)苯、2,5-二甲氧基对苯二甲醛和第3种带炔基的单体2,5-双(丙-2-炔-1-基氧基)对苯二甲醛,合成中间体[HC≡C]*_x_*-TPB-DMTP-COF,再通过叠氮-炔环加成反应(azide-ethynyl click reaction)将手性分子(*S*)-吡咯烷((*S*)-pyrrolidine)随机地锚定在COF的孔道中,得到的手性COF的结晶度和孔隙率均得以保持,且具有高的对映选择性和催化活性。除了在COFs孔道上锚定人工合成的手性小分子,直接将天然生物分子固定在COFs孔道上也可将非手性的COFs转变为手性COFs。生物分子如氨基酸、多肽、酶等拥有强的手性环境,同时具有独特的两亲性和两性离子,可与对映异构体之间产生多种相互作用,进而实现外消旋体的分离,现已被广泛用于手性固定相的制备^[8,10]^。Zhang等^[8]^利用COFs中残留的羧基与生物分子中的氨基间的共价键反应,将生物分子固定在COFs的孔道中,所得到的COFs可作为多功能和高效的手性固定相,通过与对映异构体间的静电和疏水相互作用实现对映异构体的高效分离(图1); Zhang等也考察了不同生物分子修饰后COFs的分离性能,结果发现,修饰了具有更高级结构(如酶的二级、三级和四级结构)、更多手性中心以及更强两亲性的生物分子后,COFs能展现出更突出的分离性能。

**图1:**
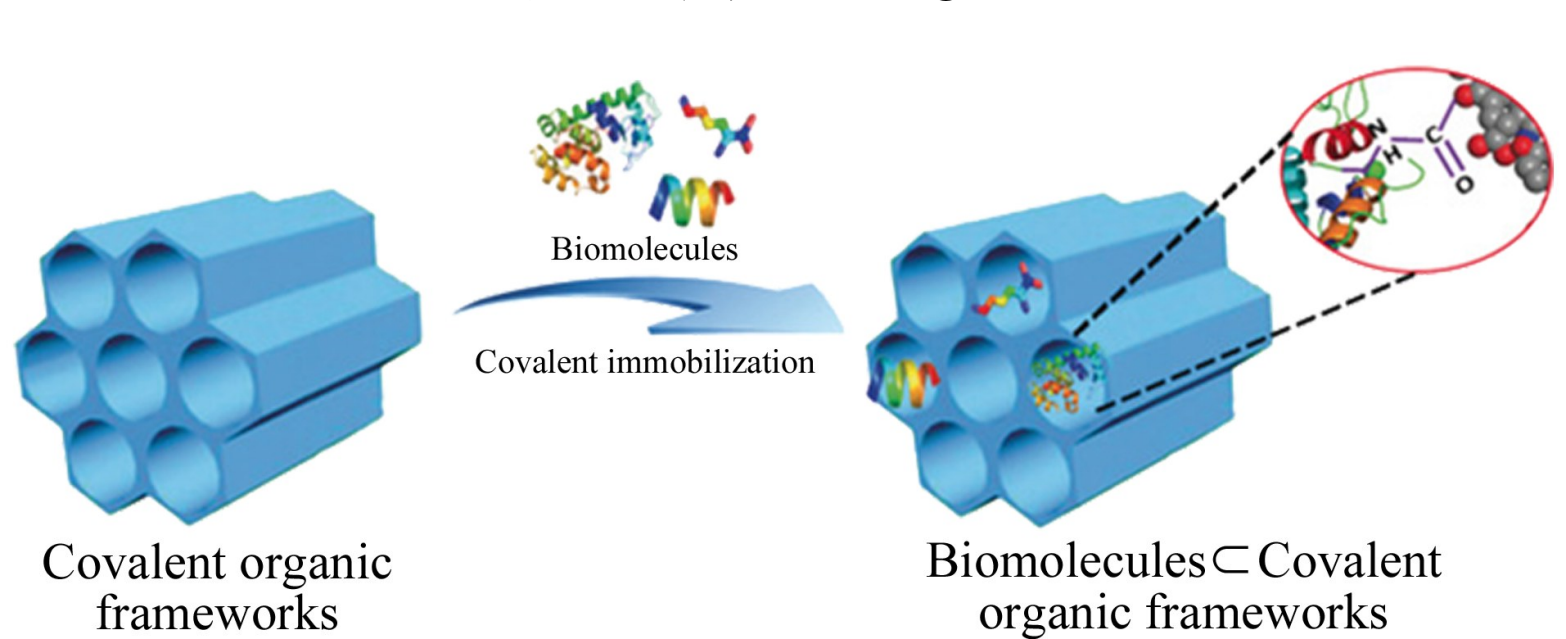
通过共价键将生物分子固定于COFs孔道内的示意图^[8]^

然而,对于采用后合成方法得到的COFs,其手性部分的分布可能是不均匀的,COFs的结晶结构也可能在后合成过程中被破坏。自下而上的合成策略是将手性中心在COFs合成前引入至单体上,使手性位点在COFs骨架中的分布更均匀、精确。此外,自下而上的合成策略也可以利用不同的手性分子或反应单体制备出多功能、多种类的手性COFs,其在手性COFs的合成中也得到了广泛认可。如图2所示,Qian等^[31]^在三醛基间苯三酚(1,3,5-triformylphloroglucinol, Tp)中引入手性(+)-二乙酰基-L-酒石酸酐((+)-diacetyl-L-tartaric anhydride, (+)-Ac-L-Ta),得到手性功能化的三醛基间苯三酚(CTp),再将其分别与3种不同的氨基单体缩合,得到高结晶度的手性COFs,即CTpPa-1、CTpPa-2和CTpBD。Cui课题组^[32]^通过手性1,2-二氨基环己烷和两种醛基单体缩合得到了两种手性COFs (CCOF 3和CCOF 4),并在缩合过程中成功锚定Zn^2+^,得到高结晶度和高比表面积的Zn(salen)-CCOF (图3)。此外,Cui等发现在COFs骨架中引入疏水性基团(如-CMe3),可以进一步提高手性COFs的稳定性。Cui课题组^[33]^将氨基单体和手性的醛基单体缩合,合成了3D手性COF((*R*,*R*)-CCOF 5),并将氨基和醛基缩合得到的亚胺键进行氧化,在维持原有3D COF的结晶度和孔隙率的情况下,制备了化学稳定性更好的手性COF ((*R*,*R*)-CCOF 6)(图4); Cui课题组^[34]^还通过自下而上的策略合成了拥有高结晶度和丰富孔隙率的手性3D COFs(CCOF 15和CCOF 16)以及高稳定性的CCOF 17和CCOF 18^[35]^。对自下而上这一策略而言,最大的挑战是手性COFs的不对称性和结晶度之间的矛盾,且手性单体合成较为困难,种类也有限,极大地限制了该方法在手性COFs合成中的应用。此外,为解决COFs的不规则形状和宽尺寸分布所带来的问题,Xu等^[36]^提出以均一的、氨基功能化的SiO_2_作为模板来生长均匀的COFs层。为实现更精准的手性识别,也有研究者提出了双手性(dual-chiral) COFs的合成策略^[37]^。

**图2:**
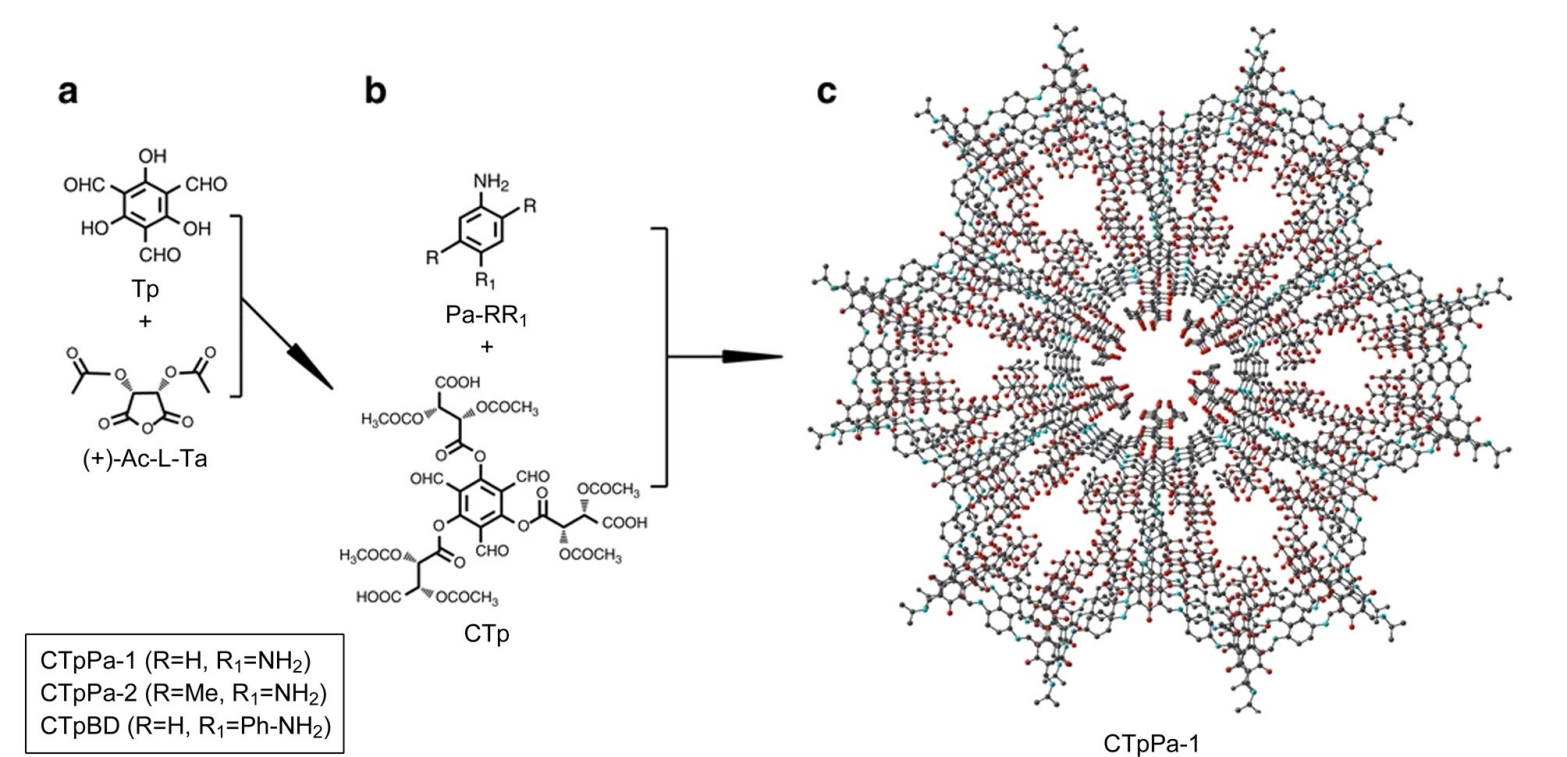
CTpPa-1、CTpPa-2和CTpBD的合成示意图^[31]^

**图3:**
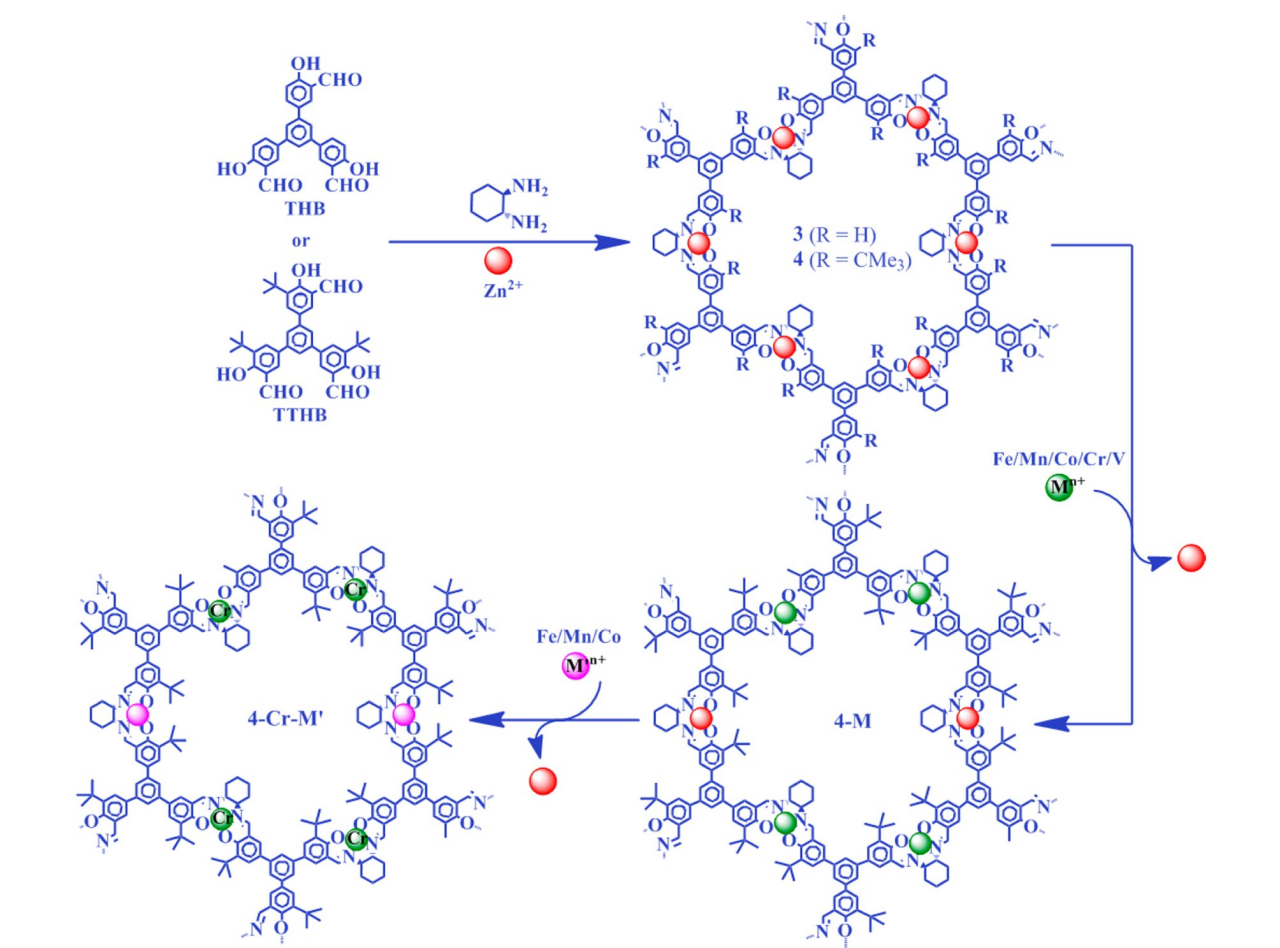
基于自下而上策略合成Zn(salen)-CCOF的示意图^[32]^

**图4:**
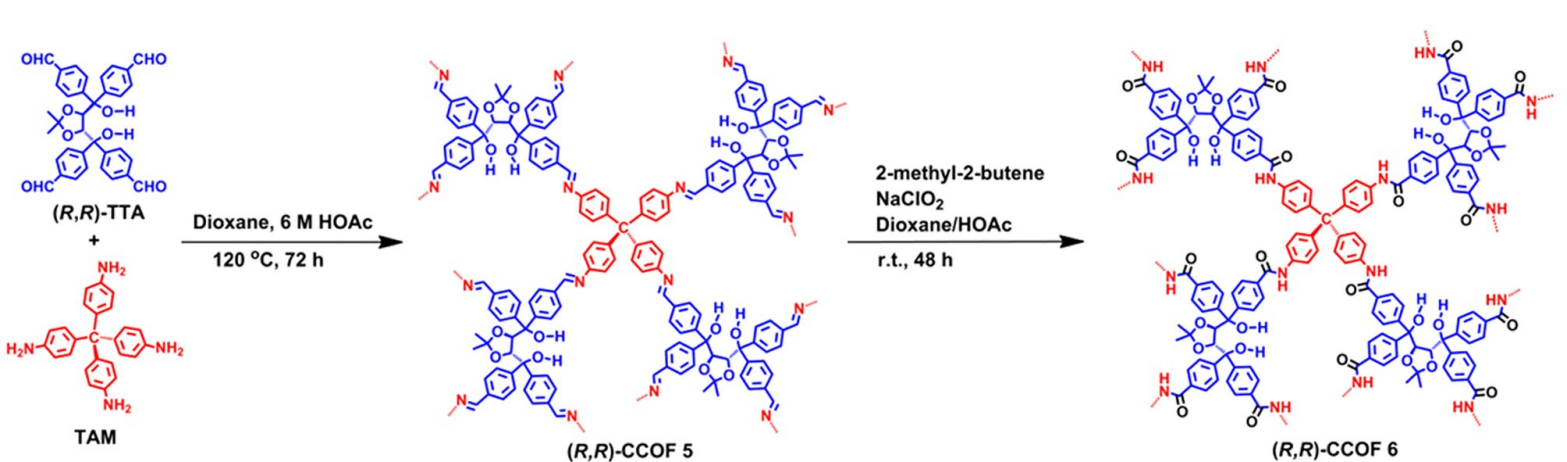
基于自下而上策略合成CCOFs的示意图^[33]^

除了后合成和自下而上的方式,通过手性诱导也可以成功制备出具有丰富手性识别位点的COFs。如图5所示,Cui课题组^[38]^在手性催化剂存在的条件下,利用三醛基间苯三酚与不同的氨基单体缩合,通过催化剂分子的构象转换,成功制备了多种手性COFs,并通过圆二色光谱证明了所合成的COFs拥有强的手性环境,这一策略为手性COFs的合成提供了新的思路。

**图5:**
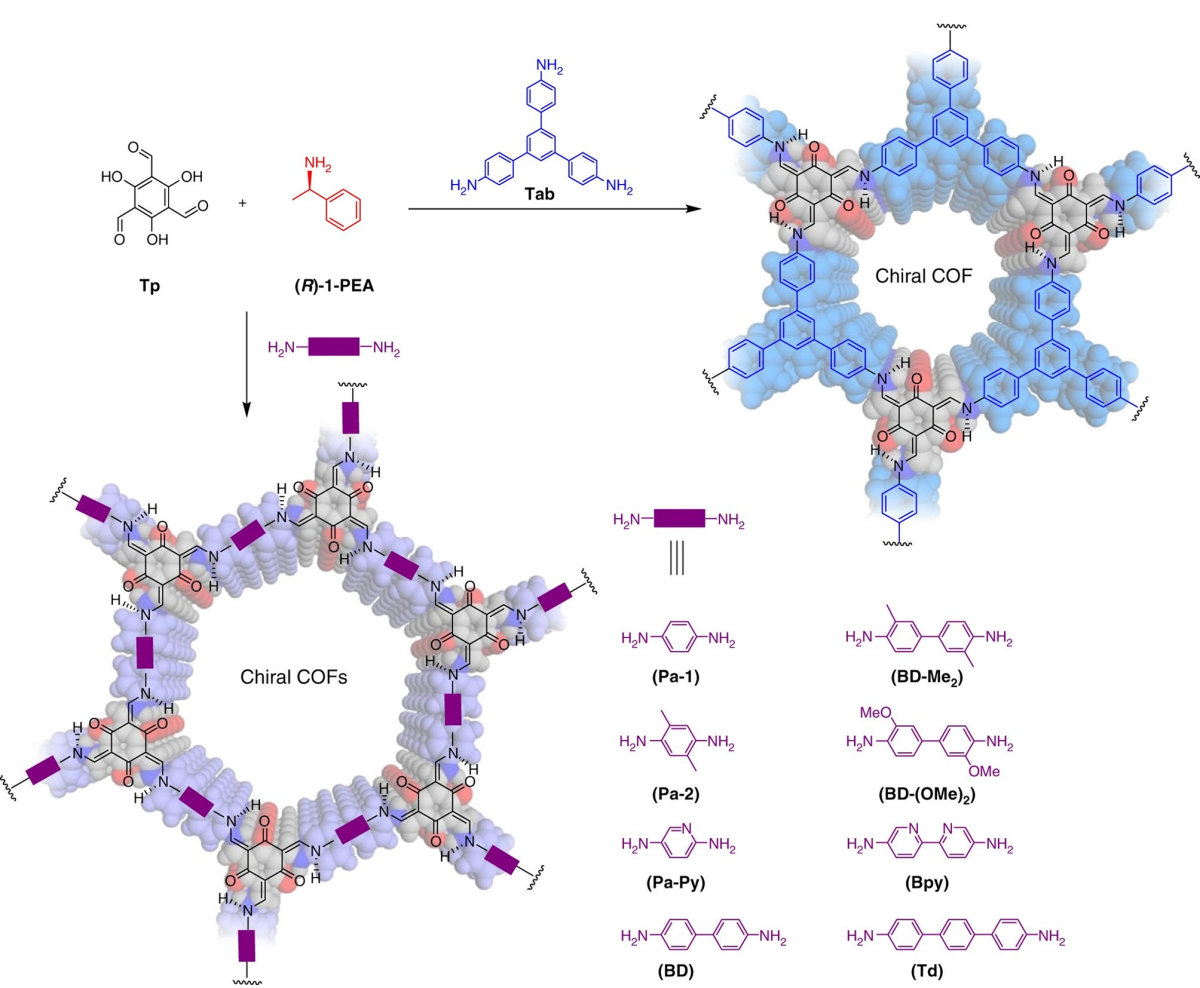
基于手性诱导策略制备手性COFs的示意图^[38]^

### 2.2 手性共价有机框架色谱柱的制备

动态涂覆法和原位生长法是制备手性COFs固定相的两种主要方式。动态涂覆法是目前普遍使用的方法^[35,39⇓⇓-42]^,其制备流程如图6所示。首先将毛细管柱用1 mol/L NaOH、纯水及0.1 mol/L HCl依次冲洗,再用纯水清洗毛细管柱直到流出溶液的pH为7,用甲醇冲洗,之后用N_2_吹干得到预处理后的毛细管柱;之后,将已制备好的COFs材料分散在合适的溶剂中,如二氯甲烷、甲醇、乙腈等,在压力泵的作用下将分散液灌入已处理好的毛细管柱中,将分散液吹出后,毛细管柱的内表面会留下一层湿润的材料涂层,用N_2_吹干,将得到的色谱柱在一定的升温程序下进行活化处理。

**图6:**
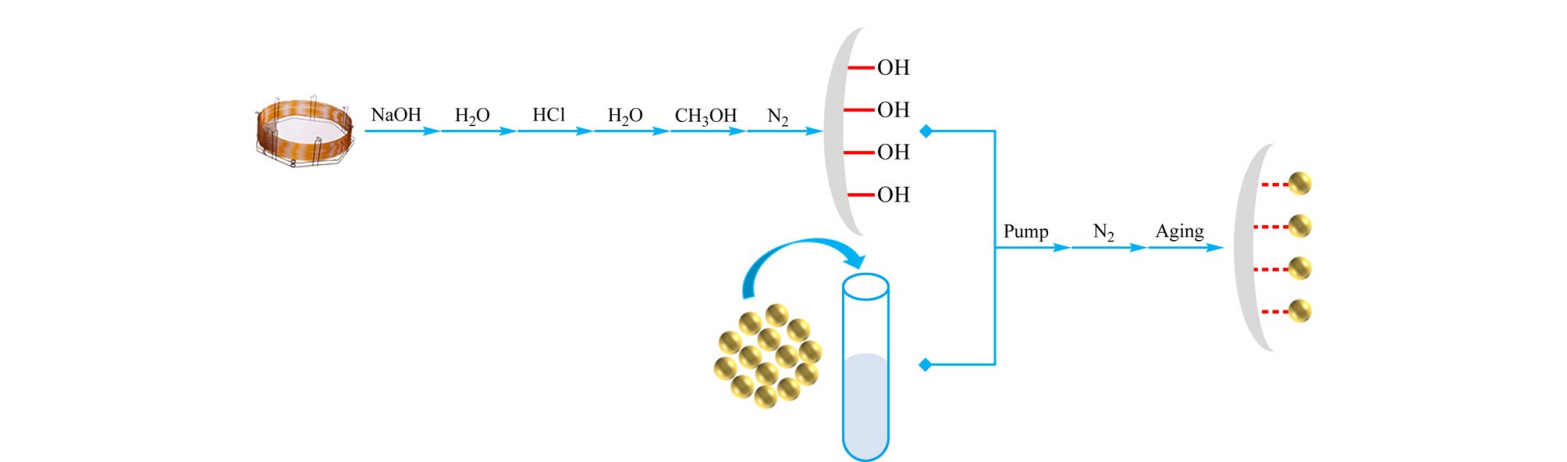
动态涂覆法制备手性COFs固定相的流程图

Tang等^[39]^将合成的手性COF分散在二氯甲烷中,通过动态涂覆的方式成功获得了COF涂覆的手性毛细管柱;Yuan等^[35]^将合成的手性CCOF用球磨机粉碎后分散在乙醇溶液中,在一定的压力下将分散液灌入15 m长、已处理好的毛细管柱中,并在毛细管柱的末端加一根2 m长的缓冲管避免溶液中的颗粒在末端积累造成柱堵塞,将毛细管柱活化后成功获得了两种手性COFs涂覆的色谱柱,涂层的厚度约为1~2 μm。

相比于动态涂覆法,原位生长法可以通过共价键作用将材料固定在色谱柱上,从而获得分布更均匀、稳定性更好的色谱固定相,其制备流程如图7所示。首先,毛细管柱分别用1 mol/L NaOH、纯水、0.1 mol/L HCl冲洗,再用纯水冲洗毛细管直至流出溶液的pH为7,最后用甲醇冲洗;随后将3-氨基丙基三乙氧基硅烷(3-aminopropyltriethoxysilane, APTES)-甲醇(1∶1, v/v)溶液灌入毛细管柱中,在40 ℃下反应过夜,用甲醇清除毛细管柱中的残留溶液,再用N_2_干燥得到氨基功能化的色谱柱;之后,将COFs的两种单体分别溶解在反应溶剂中,在0 ℃混合两种单体得到COFs的预聚合溶液,再将溶液迅速灌入毛细管中,反应一段时间后,用甲醇等溶剂移除毛细管柱中的残留物质,再在N_2_下干燥得到具有COFs涂层的色谱柱,最后在一定的升温程序下对色谱柱进行活化。Qian等^[31]^将3种手性COFs (CTpPa-1、CTpPa-2和CTpBD)均匀地分布在毛细管柱的内壁上,通过原位生长方式成功获得了3种手性色谱固定相。此外,他们用同样的方法成功将氨基功能化的3D COF均匀、紧实地固定在10 m长的色谱柱上^[43]^:首先将四(4-醛基苯基)甲烷(4-[tris(4-formylphenyl)methyl]benzaldehyde, TFPM)溶液灌入色谱柱中得到TFPM修饰的色谱柱,再与另一种单体对苯二胺(*p*-phenylenediamine, PA)溶液混合后,将TFPM-PA的预聚合溶液迅速灌入TFPM修饰的色谱柱中,反应一段时间后即可成功将TFPM-PA通过共价键固定在色谱柱上;之后,向固定了TFPM-PA的色谱柱中灌入3,3'-二氨基联苯胺(3,3'-diaminobenzidine, BD-NH_2_)的二甲基亚砜溶液,通过构建块互换的方式,制备出氨基功能化的3D COF(JNU-5);然而,由于COFs的两种单体预聚合速度快,易快速生成沉淀,这给原位生长策略带来了一定的挑战。

**图7:**
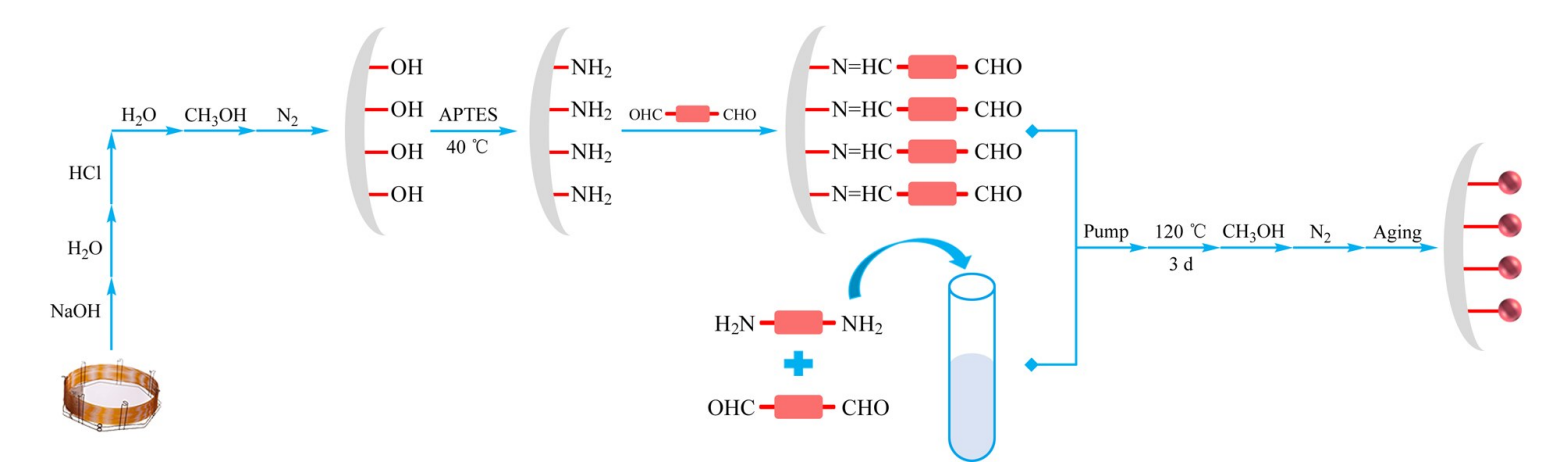
原位生长法制备手性COFs固定相的流程图

### 2.3 手性共价有机框架应用于色谱分离

手性COFs的热稳定性好,孔隙丰富,可通过尺寸匹配效应及与分析物之间的多种相互作用(如氢键、静电、*π-π*和C-H…*π*相互作用等)来实现多种同系物、同分异构体以及手性混合物的分离。

Qian等^[31]^通过原位生长法制备的3种手性COFs色谱柱(CTpPa-1、CTpPa-2和CTpBD (30 m×0.32 mm))对(±)-1-苯乙醇、(±)-1-苯基-1-丙醇、(±)-柠檬烯和(±)-乳酸甲酯均表现出优异的分离性能(图8),整个分离过程不超过5 min,而商用的手性色谱柱*β*-DEX 225和Cyclosil B则不能分开(±)-1-苯基-1-丙醇及(±)-柠檬烯;3种自制的手性COFs色谱柱对这4类外消旋体的选择因子(*α*)为1.88~2.68,分离度(*R*)为1.56~2.48,*α*和*R*均明显优于两种商用的手性色谱柱;此外,为了进一步探究手性COFs对对映异构体的保留作用和手性识别能力,Qian等还研究了自制手性色谱柱在对映异构体混合物分离过程中的热力学参数。结果发现,与(-)-对映异构体相比,(+)-对映异构体具有更小的熵变(Δ*S*)和焓变(Δ*H*),表明(+)-对映异构体在手性COFs固定相中更加有序,且与手性COF间的相互作用更强,从而使自制的手性COFs固定相具有更高的对映异构体分离效率。环糊精(cyclodextrin, CD)及其衍生物具有丰富的氨基官能团、手性位点以及独特的空腔结构,Tang等^[39]^将七(6-氨基-6-去氧)-*β*-环糊精(heptakis(6-amino-6-deoxy)-*β*-CD, Am7CD)作为手性氨基单体,与对苯二甲醛(terephthalaldehyde, TPA)缩合得到具有强手性的*β*-CD-COF,再通过动态涂覆法制得*β*-CD-COF色谱柱(20 m×0.25 mm),实现了烷烃(*n*-C_12_~*n*-C_17_)、醇类同系物(*n*-C_8_-OH~*n*-C_12_-OH)、脂肪酸甲酯类混合物的分离,且色谱峰不存在拖尾现象;在Grob混合物(包括甲基癸酸酯、甲基十一酸酯、甲基十二酸酯、癸烷、十一烷、十二烷、辛醇-1、壬醛、2,3-丁二醇、2,6-二甲苯胺、2,6-二甲苯酚、二环己基胺、2-乙基己酸)的分离分析中,除2,6-二甲基苯酚和2,6-二甲基苯胺的色谱峰有部分重叠外,其他物质均得到了有效的分离;同时,*β*-CD-COF色谱柱对烷基苯的同系物(甲苯、乙苯等)及位置异构体(硝基苯胺、二氯苯等)也表现出较好的分离效果。进一步评估了该手性色谱柱的手性识别能力,结果表明,该色谱柱对10个外消旋体(如DL-组氨酸衍生物、DL-谷氨酰胺衍生物、DL-丝氨酸衍生物等)均表现出较好的分离能力,*α*均大于1,*R*最大可达1.48,几乎接近完全分离的标准(*R*≥1.5)。Yuan等^[35]^通过动态涂覆法制备的CCOF手性色谱柱(15 m×0.25 mm)对多种外消旋体(包括谷氨酸、乙酸乙酯、苯丙氨酸和2-甲基戊醛等)也具有较好的分离性能(图9),其*R*最大可分别达1.51、1.94、0.68和1.13。由此可见,手性共价有机框架作为手性色谱固定相在对映异构体分离方面具有广阔的应用前景。

**图8:**
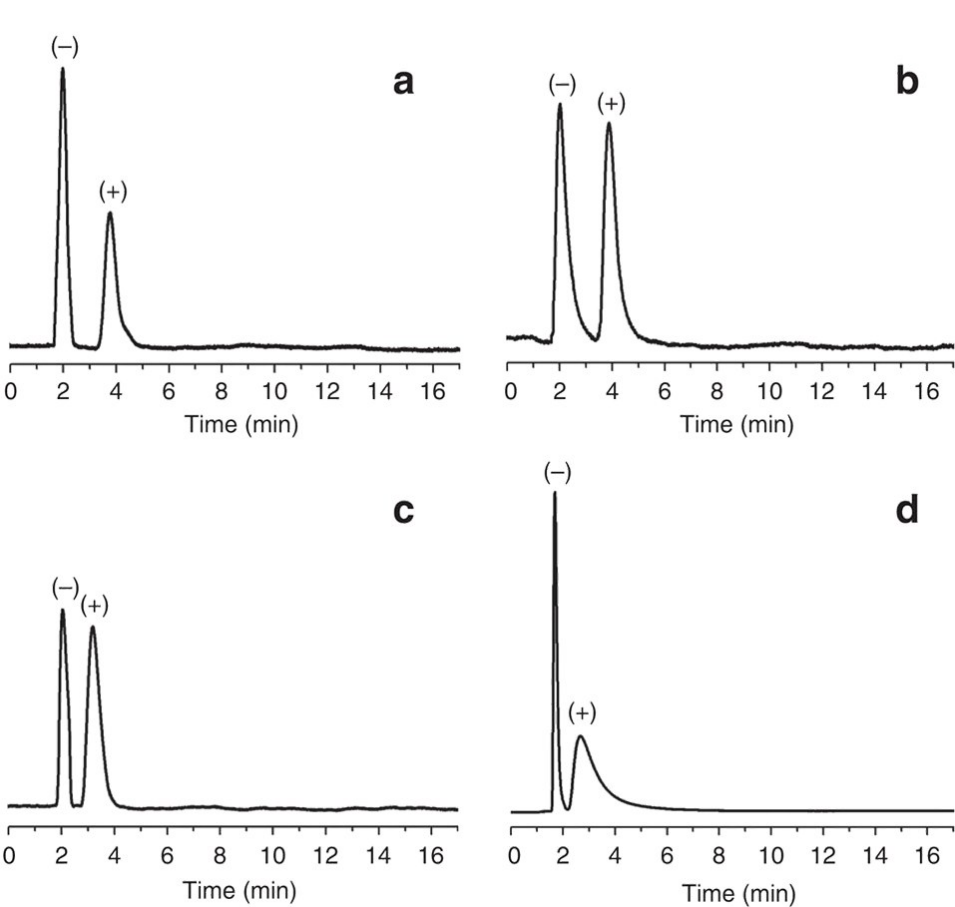
通过原位生长法制备的手性CTpPa-1色谱柱对(a)(±)- 1-苯乙醇、(b) (±)-1-苯基-1-丙醇、(c) (±)-柠檬烯和(d) (±)-乳酸甲酯的气相色谱分离图^[31]^

**图9:**
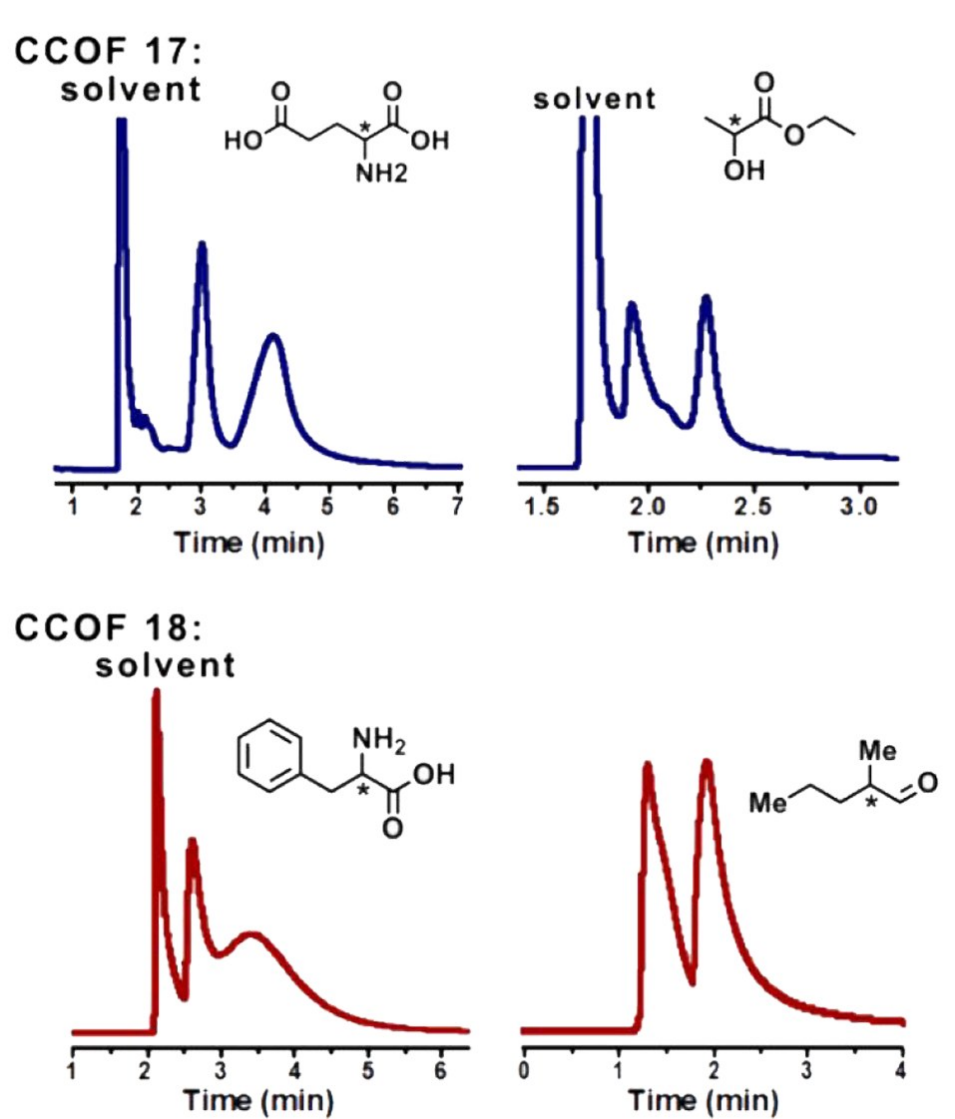
通过动态涂覆法制备的手性CCOF色谱柱对谷氨酸、乙酸乙酯、苯丙氨酸和2-甲基戊醛的气相色谱分离图^[35]^

## 3 手性金属有机框架

MOFs也是一类新兴的多孔有机框架材料,其由金属离子或金属簇作为节点、多种有机配体作为连接单元,自组装构建而成。MOFs中存在可利用的开放孔结构,并具有结晶度高、孔隙率丰富、孔径可调、合成可控等优点。手性MOFs作为MOFs的一个重要分支,既具有MOFs的多样结构和功能特性,也拥有较强的手性环境,已在对映异构体分离领域表现出重要的潜力,吸引着越来越多的关注。手性MOFs固定相应用于色谱分离的关键在于手性MOFs的合成,在3.1~3.3节将对手性MOFs的合成、手性MOFs色谱柱的制备及手性MOFs在色谱分离中的应用进行逐一介绍。

### 3.1 手性金属有机框架材料的合成

与手性COFs的合成方式相似,手性MOFs的合成主要包括后合成和自下而上两种方式。后合成是合成手性MOFs的常见方式。首先通过控制反应条件(如反应温度、时间、金属盐和配体的比例以及加入调制剂等方式)调节MOFs的形貌和尺寸,从而获得形貌均匀、尺寸可控的MOFs晶体,再通过后修饰策略将手性中心锚定在MOFs的框架上。我们课题组^[44]^通过改变调制剂2-甲基咪唑的用量及反应温度合成了尺寸约为5 μm、形貌均匀的Co-MOF-74颗粒,之后再将中性的L-酪氨酸(L-tyrosine, L-Tyr)固定在Co-MOF-74的框架中(图10)。红外光谱、核磁共振氢谱和圆二色光谱均表明L-Tyr已成功引入Co-MOF-74的框架中。X射线衍射测试结果表明,引入L-Tyr后,Co-MOF-74的框架结构仍存在。Ding等^[45]^将乳糖醛酸(lactobionic acid, LA)作为手性位点,引入沸石咪唑MOF-90 (ZIF-90)中,制备了具有手性拆分能力的MOF材料。Sun等^[46]^将羧甲基化的*β*-环糊精(carboxymethyl-*β*-cyclodextrin, CM-*β*-CD)通过后合成策略引入MOF-199 (也称Hong Kong University of Science and Technology-1, HKUST-1)中,并将其用于对映异构体的分离。

**图10:**
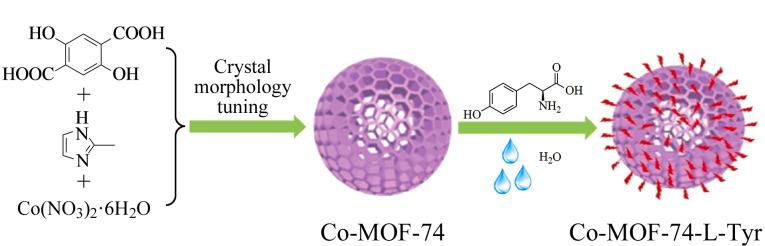
Co-MOF-74-L-Tyr的合成示意图^[44]^

基于自下而上策略直接合成手性MOFs通常需要使用手性配体或手性结构单元,如单一手性的有机酸分子和自然存在的氨基酸分子,充分利用其能与金属离子配位的羧基或氨基,构建强手性的MOFs材料。Ma等^[47]^通过同时加入L-苯丙氨酸(L-phenylalanine, L-Phe)、1,2-双(4-吡啶)乙烯(1,2-bis(4-pyridyl)ethene, bpe)及Zn(Ⅱ)盐溶液一步构建了手性[Zn_2_(L-Phe)_2_(bpe)_2_]*_n_*,并利用X射线衍射对其单晶结构进行解析。结果表明,Zn(Ⅱ)与两个分别来自不同bpe的N原子、一个来自L-Phe的N原子以及一个来自不同L-Phe分子的O原子配位,从而得到扭曲的三角双锥结构。Mon等^[48]^通过使用L-组氨酸(L-histidine)的衍生物作为手性配体与Cu(Ⅱ)配位,成功制备了手性MOF。逐层生长法(layer-by-layer growth)也可用于控制MOF的形貌和尺寸,Yu等^[49]^在形貌均匀的羧基功能化SiO_2_微球上生长D-his-ZIF-8,从而获得形貌均匀、窄尺寸分布的核壳结构微球,并将其作为手性固定相用于对映异构体的分离。

此外,在没有手性试剂引入时,通过对称断裂结晶(symmetry breaking crystallization)也可以获得手性结构,即非手性前驱体在外部刺激(如搅拌、化学处理或研磨等技术)下结晶得到手性MOFs。Yu等^[50]^发现通过改变反应溶剂和温度,能获得单一手性的两种MOF (1P-H_2_O([(Co)_6_(L)_6_(H_2_O)])·*x*H_2_O和1M-NH_3_([(Co)_6_(L)_6_(NH_3_)])·*x*H_2_O),并通过单晶X射线衍射法和圆二色光谱证实了它们的对映异构性。但是通过该方法获得的手性中心通常是不规则且难以预测的,因此基于逐层生长法获得的手性MOFs目前还鲜有报道。

### 3.2 手性金属有机框架色谱柱的制备

手性MOFs色谱柱的制备方式主要为动态涂覆法和原位生长法。动态涂覆法是将手性MOFs固定在毛细管柱上最常用的方式。毛细管柱分别用1 mol/L NaOH、超纯水、0.1 mol/L HCl进行处理,再用超纯水冲洗毛细管柱直至流出溶液的pH为7,再用甲醇溶液冲洗,最后在N_2_气氛下干燥毛细管柱。将1 mg左右的材料均匀分散在1 mL乙醇等溶剂中,在一定压力下将混合溶液灌入毛细管柱中,溶液流出毛细管柱后将在毛细管柱内表面留下一层湿润的材料涂层,之后将毛细管柱在N_2_气流下干燥过夜,并在一定的升温程序下进行活化。Kou等^[51]^先通过后合成方法在氨基修饰的MIL-101(Al)-NH_2_上共价接枝了5种不同的手性分子,从而制得5种手性MOFs,再通过动态涂覆的方式获得了手性MOFs色谱柱。

原位生长法是指通过单层的自组装方式(self-assembled monolayer, SAM)重复多次后获得MOF薄层。Wu等^[52]^先依次用1 mol/L NaOH、超纯水、0.1 mol/L HCl冲洗色谱柱2 h、30 min、2 h,再用超纯水冲洗毛细管柱直至流出溶液的pH为7,再用甲醇溶液冲洗30 min,最后在N_2_气氛下干燥毛细管柱,从而暴露出毛细管柱内表面上的羟基;之后将丁二酸酐和APTES的混合溶液迅速灌入毛细管柱中,将毛细管柱两端密封后在30 ℃下反应一段时间,将溶液冲出后用乙醇冲洗并用N_2_干燥,得到羧基修饰的毛细管柱;之后将2-咪唑甲醛、甲酸钠和Zn(NO_3_)·6H_2_O的混合溶液灌入色谱柱中,在室温下反应一段时间后将溶液冲出并用甲醇冲洗,该步骤重复两次后在N_2_气氛下干燥,并在一定的升温程序下进行活化。

### 3.3 手性金属有机框架应用于色谱分离

已有报道证明手性MOFs可作为手性色谱固定相实现对映异构体的有效分离。Xie等^[53]^合成了手性MOFs,并通过动态涂覆法将其固定在毛细管柱(2 m×75 μm)上作为气相色谱固定相,实现了11种外消旋物的分离。Xie等^[54]^通过动态涂覆法制备了手性MOFs气相色谱柱(2 m×75 μm),实现了柠檬烯、1-苯基-1,2-乙二醇、天冬氨酸、脯氨酸和亮氨酸5种外消旋物的分离,其*R*最大可达1.38;同时,将气相色谱与液相色谱、毛细管电色谱的分离结果相比较发现,分离条件、MOFs的组分和拓扑结构均会影响对映异构体的分离性能。Kou等^[51]^以氨基功能化的MIL-108(Al)-NH_2_作为母体MOF,通过后合成的方式分别接上5种不同的手性分子,合成出的MOFs具有优异的稳定性和丰富的识别位点;再通过动态涂覆法将其制备为手性MOFs色谱柱(30 m×0.25 mm),实现了2-甲基-2,4-戊二醇、1,2-戊二醇、香茅醛、扁桃腈等对映异构体的分离;与商用的手性色谱柱相比,自制的手性色谱柱具有更优的对映异构体分离性能,其*R*最大可达1.67。由此可见,合理设计手性MOFs固定相的合成方案,可以有效实现对映异构体的分离。

## 4 手性多孔有机笼和手性金属有机笼

POCs是由具有永久孔隙的离散分子笼通过弱的分子间作用力组装形成,与通过共价键或配位键组装得到的COFs和MOFs不同的是,POCs在普通的有机溶剂中可溶,其结构易改性,也易与其他材料一起形成复合材料,同时也便于通过静电涂覆的方式让其在毛细管柱内成膜,因此POCs在分离领域中吸引了广泛的关注^[16]^。到目前为止,多种手性POCs已被成功制备,并用于对映异构体的分离中。Kewley等^[55]^通过间苯三甲醛和(*R*,*R*)-1,2-环己烷二胺在三氟乙酸催化下缩合得到手性POCs(CC3-R),之后将得到的CC3-R均匀分散液静电涂覆至毛细管柱上,用于分离多种线性烷烃、手性醇及手性胺。Zhang等^[56]^将CC3-R溶解在聚硅氧烷(OV-1701)中,通过静电涂覆的方式制备了气相色谱固定相。结果表明,与商用的*β*-DEX 120和Chirasil-L-Val色谱柱相比,所制备的色谱柱具有更好的对映异构体分离性能。此外,Li等^[57]^也合成了一种羟基功能化的手性POCs,采用相同的方式制备成气相色谱柱之后,成功应用于多种同分异构体和外消旋物的分离。Wang等^[58]^还成功在硫醇功能化的二氧化硅上通过点击反应固定了手性POCs(NC1-R),用于多种外消旋体的分离。目前大部分通过醛胺缩合得到的手性POCs中,亚胺键的键能较低,易受亲核试剂的进攻,在潮湿环境中材料结构易坍塌。为了解决这一问题,Cui等^[59]^提出将亚胺键转变为氨基,并采用带有疏水性芳香环骨架的单体,合成了稳定的手性芳香笼(PAC 1-S和PAC-R),用于对映异构体的分离。

MOCs是由金属离子或金属簇与有机配体通过配位自组装形成的离散笼状分子构建而成的,其与环糊精衍生物和POCs具有相似的性质。目前已经报道了较多结构新颖的手性MOCs材料。Xie等^[60]^通过将Zn(CH_3_COO)_2_·2H_2_O的甲醇溶液在搅拌下添加到手性[3+3]大环配体(ligand, L)的甲醇溶液中,经加热、冷却后放置在冰箱中过夜,成功制备了手性的MOC [Zn_3_L_2_];将[Zn_3_L_2_]与OV-1701混合,通过静电涂覆制备了手性的MOC气相色谱柱,实现了多种同系物和对映异构体混合物的分离。

## 5 手性氢键有机框架材料

HOFs是有机分子通过分子间氢键相互作用来构建的,与MOFs和COFs相比,HOFs的框架结构更灵活,也可以通过简单的重结晶回收再利用,同样在分离领域中被广泛研究。2014年,手性HOFs首次成功制备,Li等^[61]^采用可形成强氢键作用的2,4-二氨基三嗪基(2,4-diaminotriazinyl, DAT)和带有不对称中心的1,1'-双-2-萘酚(1,1'-bi-2-naphthol, BINOL),成功合成了手性HOF-2,实现了对小分子对映异构体的有效分离。Veselovsky等^[62,63]^用相同的氨基酸分别取代不同的二芳基乙炔二羧酸,将得到的分子进行自组装,成功制备了新型的HOFs(ZIOC-1和ZIOC-2),所合成的HOFs框架灵活性高,可随环境温度和湿度的改变而改变。Wang等^[64]^成功将HOF-2颗粒涂覆在气相色谱毛细管柱上,实现了多种烷烃、醚类、烷基苯类混合物、同分异构体和外消旋体的分离。

## 6 总结和展望

手性有机框架材料具有独特的结构、丰富的手性识别位点、高的比表面积和易于改性等特点,在对映异构体分离领域有很大的应用潜力。后合成和自下而上策略是制备手性有机框架材料的主要方式;其中,前者是引入手性部分最常用的方式,但手性位点分布不够均匀;后者手性位点分布更均匀,但手性单体难合成,且合成过程中还需要考虑结构规整性的问题。因此,目前手性有机框架材料的合成还存在一定的挑战。动态涂覆和原位生长等方法可将有机框架材料制备成手性色谱柱涂层,目前动态涂覆法是最常用的方式,但对涂层材料的分散性要求较高;原位生长的方式可以获得更均匀、性能更稳定的涂层,但由于色谱柱前处理较复杂,且手性有机框架材料合成困难等问题还未被广泛采用。此外,手性涂层用于识别对映异构体的机制主要有主-客体相互作用、氢键、*π-π*相互作用、空间位阻、范德华力等,其机理还缺乏深入的探究。随着手性有机框架材料合成方法的突破及手性识别机理的深入研究,手性有机框架材料将可能成为手性材料领域一个十分重要的分支,在对映异构体分离领域展现出更突出的优势,并成功应用于大规模的手性物质分离分析和生产。
